# Primary care management for patients receiving long-term antithrombotic treatment: A cluster-randomized controlled trial

**DOI:** 10.1371/journal.pone.0209366

**Published:** 2019-01-09

**Authors:** Andrea Siebenhofer, Lisa-Rebekka Ulrich, Karola Mergenthal, Andrea Berghold, Gudrun Pregartner, Birgit Kemperdick, Sylvia Schulz-Rothe, Sandra Rauck, Sebastian Harder, Ferdinand Michael Gerlach, Juliana Johanna Petersen

**Affiliations:** 1 Institute of General Practice and Evidence-based Health Services Research, Medical University of Graz, Graz, Austria; 2 Institute of General Practice, Goethe-University Frankfurt am Main, Frankfurt am Main, Germany; 3 Institute for Medical Informatics, Statistics and Documentation, Medical University of Graz, Graz, Austria; 4 Institute of Clinical Pharmacology, Goethe-University Frankfurt am Main, Frankfurt am Main, Germany; Maastricht University Medical Center, NETHERLANDS

## Abstract

**Purpose:**

To examine whether applying case management in general practices reduces thromboembolic events requiring hospitalization and major bleeding events (combined primary outcome). Secondary endpoints were mortality, frequency and duration of hospitalization, severe treatment interactions, adverse events, quality of anticoagulation, health-related quality of life and intervention costs, patients’ assessment of chronic illness care, self-reported adherence to medication, GP and HCA knowledge, patient knowledge and satisfaction with shared decision-making.

**Methods:**

Cluster-randomized controlled trial undertaken at 52 general practices in Germany with adult patients with a long-term indication for oral anticoagulation. The complex intervention included training for healthcare assistants, information and quality circles for general practitioners and 24 months of case management for patients. Assessment was after 12 and 24 months. The intention-to-treat population included all randomized practices and patients, while the per-protocol analysis included only those that received treatment without major protocol violations.

**Results:**

The mean (SD) age of the 736 patients was 73.5 (9.4) years and 597 (81.1%) had atrial fibrillation. After 24 months, the primary endpoint had occurred in 40 (11.0%) intervention and 48 (12.9%) control patients (hazard ratio 0.83, 95% CI 0.55 to 1.25; *P* = .37). Patients’ perceived quality of care, their knowledge, and HCAs’ knowledge, had improved significantly at 24 months. The other secondary endpoints did not differ between groups. In the intervention group, hospital admissions were significantly reduced in patients that received treatment without major protocol deviations.

**Conclusions:**

Even though the main outcomes did not differ significantly, the intervention appears to have positively influenced several process parameters under ‘real-world conditions’.

## Introduction

Oral anticoagulation (OAC) has been shown to be highly effective in preventing thromboembolic complications in patients for whom it is indicated. In antithrombotic treatment, vitamin K antagonists (VKAs) have been the agent of choice for several decades. VKAs carry a considerable risk of adverse thromboembolic and bleeding events, particularly in the case of dose deviations when international normalized ratio (INR) values are outside the target range [1]. However, when patients are able to perform self-management, thromboembolic events and all-cause mortality are less frequent, and treatment-related quality of life rises [2,3].

Although subject to a number of concerns [4], direct oral anticoagulants (DOACs) are considered an effective alternative to VKAs in the long-term treatment of anticoagulation, and prescriptions have risen strongly since they were approved in 2011 [5].

Patients taking oral anticoagulation often suffer from multiple chronic conditions and have complex health care needs. Understanding and managing complex patients is a quintessential feature of primary care [6]. Organizing services to improve care for these patients has been identified as a priority for the health care system, and especially for primary care research [7].

In Germany, management of patients taking OAC is typically carried out by general practitioners (GPs) in their practices. They generally employ one or more healthcare assistants (HCAs), whose role is comparable to medical assistants in the United States. In small primary care settings, resources are often limited and extensive collaborative models may be difficult to implement. The effectiveness of programs that expand the role of healthcare assistants in primary care to include chronic care services, such as case management in patients with depression [8,9] and chronic heart failure [10] have shown positive effects.

The aim of this study was to improve antithrombotic management in primary health care by having a healthcare assistant perform major elements of case management, and testing its effectiveness in reducing thromboembolic events requiring hospital admission, and major bleeding events.

## Methods

### Study design and population

The primary care management for optimized antithrombotic treatment (PICANT) study was an open cluster-randomized controlled trial undertaken at 52 general practices in Germany. The trial was registered at ISRCTN41847489 and approved by the ethics committee (E 191/11) of Frankfurt University Hospital on June 26, 2012. The study protocol and the practice recruitment process is described in detail elsewhere [11,12]. In brief, we identified potentially eligible practices from a list provided by the Association of Statutory Health Insurance Physicians (mandatory registration of GP practices). As the list only contains the names and addresses of GPs, we mailed information on the trial to 568 randomly selected practices (6% of all registered practices in 2012) and invited them to participate. Inclusion criteria were only checked for those who were interested in participation. Practice recruitment was stopped when 52 practices had enrolled, even though further practices were interested in participating.

Each participating practice was visited after practice recruitment but before cluster randomization, and asked to generate a screening list of potentially eligible patients. The practice software was used to generate the lists, and each practice was advised by study team members based on predefined instructions and search terms [11]. The GPs then checked the lists and deleted cases of patients that had only been seen occasionally, or had died in the meantime. Inclusion criteria were then assessed by the GP and study team for 30 randomly selected patients from the list, with the aim of recruiting 15 patients per practice for the study. A documentation sheet was filled in for each screened patient. To avoid selection bias, the order of the patients assessed for eligibility was chosen using the random number generator function in Microsoft Excel.

Patients were eligible for inclusion if they were ≥ 18 years of age, had a long-term indication for oral anticoagulation based on the guidelines valid at the time, and were prescribed coumarins, antiplatelet therapies, or the DOACs that were on the market when the study began (dabigatran, rivaroxaban). Exclusion criteria were dementia, diseases resulting in a life expectancy of < 6 months, psychosis, severe sight disorders or auditory defects, alcohol- or drug abuse, residence in institutions that did not allow study participation, and a lack of German language skills.

We obtained written informed consent from participants. The assessment occurred three times: at baseline and at follow-ups after 12 and 24 months.

### Randomization and masking

After the baseline assessment had been completed, a member of the Institute of General Practice that had no further involvement in the study used the web-based randomization tool “Randomizer for Clinical Trials” (http://www.randomizer.at) to consecutively and randomly allocate practices to the intervention or routine care arm in a ratio of 1:1. Randomization was stratified according to the number of inhabitants in the postal area where the practice was located and using permuted blocks of size 8. The statisticians were blinded to group assignment during the analysis [11].

### Intervention

Before randomization, all practices were provided with the evidence-based “Anticoagulation” guideline for general practitioners prepared by the Guideline Group of the German state of Hesse, and a standardized information brochure for patients issued by the German College of General Practitioners and Family Physicians [11].

The complex intervention included the provision of additional tools and guidelines to GPs and their practice teams (for intervention details see S1 Table). During an interactive one-day workshop, HCAs were trained to carry out case management and educate patients [11], assess adherence to medication and symptoms in patients, and to regularly monitor them using the Coagulation-Monitoring-List (Co-MoL) [13]. Furthermore, HCAs were taught to encourage patients to perform self-management whenever feasible. GPs and HCAs were both provided with information materials and fact sheets on phenprocoumon, dabigatran, rivaroxaban, acetylsalicylic acid, and clopidogrel. In addition, we telephoned GPs immediately after randomization and provided them with further information on case management. Quality circles to discuss the practical problems involved in anticoagulation and preparing individual case reports took place three times during the course of the trial (for further details on the intervention please see S1 Table and Siebenhofer et al. [11]). To help cover increased staff costs, intervention practice teams received €50 per enrolled patient and assessment.

For the duration of the trial, the patients in the control group continued to receive treatment-as-usual from their GPs, meaning that except the fact that GPs received the “Anticoagulation” guideline for general practitioners before randomization, no additional advice was given and no regular monitoring visits took place. After the trial had ended, a similar HCA workshop to that provided in the intervention group was offered to participating practices. A financial incentive of €25 per enrolled patient and assessment was granted.

### Endpoints

The primary patient-relevant endpoint was the combination of all thromboembolic events requiring hospitalization and major bleeding complications (if more than one event occurred in a patient, only the earliest event was considered; for the exact definition of major bleeding and thromboembolic events see study protocol [11]). Two external, independent, and blinded reviewers cross-checked primary endpoints by assessing hospital discharge letters and case report forms (TG and MS).

The following key secondary endpoints were evaluated: all-cause and cause-related mortality rates, frequency and duration of hospitalization, number of recurrent strokes (ischemic and hemorrhagic stroke), major bleeding and thromboembolic complications (counting all events), number of patients with at least one potentially severe treatment interaction, total number of potentially severe treatment interactions involving oral anticoagulants, number of adverse events, quality of anticoagulation (i.e. time within therapeutic range) [14], health-related quality of life (EQ-5D) [15], and costs from the payer’s perspective (German statutory health insurance).

Further secondary outcomes were investigated to explain factors that may have influenced the intervention’s effectiveness: patients’ assessment of chronic illness care (PACIC short version) [16], self-reported adherence to medication (questionnaire by Morisky; sum score 0–4, with lower scores indicating lower adherence) [17], GP and HCA knowledge (self-developed knowledge questionnaire, sum score 0–12), patient knowledge (questionnaire developed by Hua, sum score 0–13) [18], and satisfaction with shared decision-making (Man-Song Hing test) [19].

### Statistical analyses

Sample size was calculated using the primary combined endpoint. We anticipated an event rate of 15% in the routine care and 7.5% in the intervention group, an intra-cluster correlation coefficient (ICC) of 0.01, and 15 patients per practice. Using a chi-square test, 317 patients per group were required to detect the difference in event rates at a 5% significance level and a power of 80%. To allow for patient withdrawals and practice loss, we aimed to include 23 general practices and 345 patients per group. The sample size calculation was performed using nQuery Advisor 7.0. For the primary endpoint, the time from randomization to first thromboembolic event requiring hospitalization, or major bleeding complication, was analyzed using a Cox proportional hazards model with robust sandwich estimates to account for clustering. Secondary survival endpoints were likewise analyzed. Mixed-effects regression models with practices as random effects were used to analyze all remaining outcomes—linear models for continuous data, logistic models for binary data and Poisson models for count data. Accordingly, results are either hazard ratios (HR), mean differences (MD), odds ratios (OR) or risk ratios (RR), each reported with a 95% confidence interval (CI). We also present ICCs.

The primary analysis was of the intention-to-treat (ITT) population, including all randomized practices and their patients. We also performed a per-protocol (PP) analysis that included only those practices and patients that received treatment without major protocol violations. Furthermore, in a modified intention-to-treat analysis (mITT), patients switching to the new antithrombotic treatment were censored at the time of switching. We performed sensitivity analyses for survival outcomes using the date of the first HCA training session as an alternative starting point in the intervention group, and adding the median time between randomization and the beginning of intervention group training to the start date in the control group. We also performed subgroup analyses for gender.

We used SAS 9.4 and R, version 3.3.3, for the statistical analyses. A p-value of less than 5% was considered significant.

## Results

**Fig 1:**
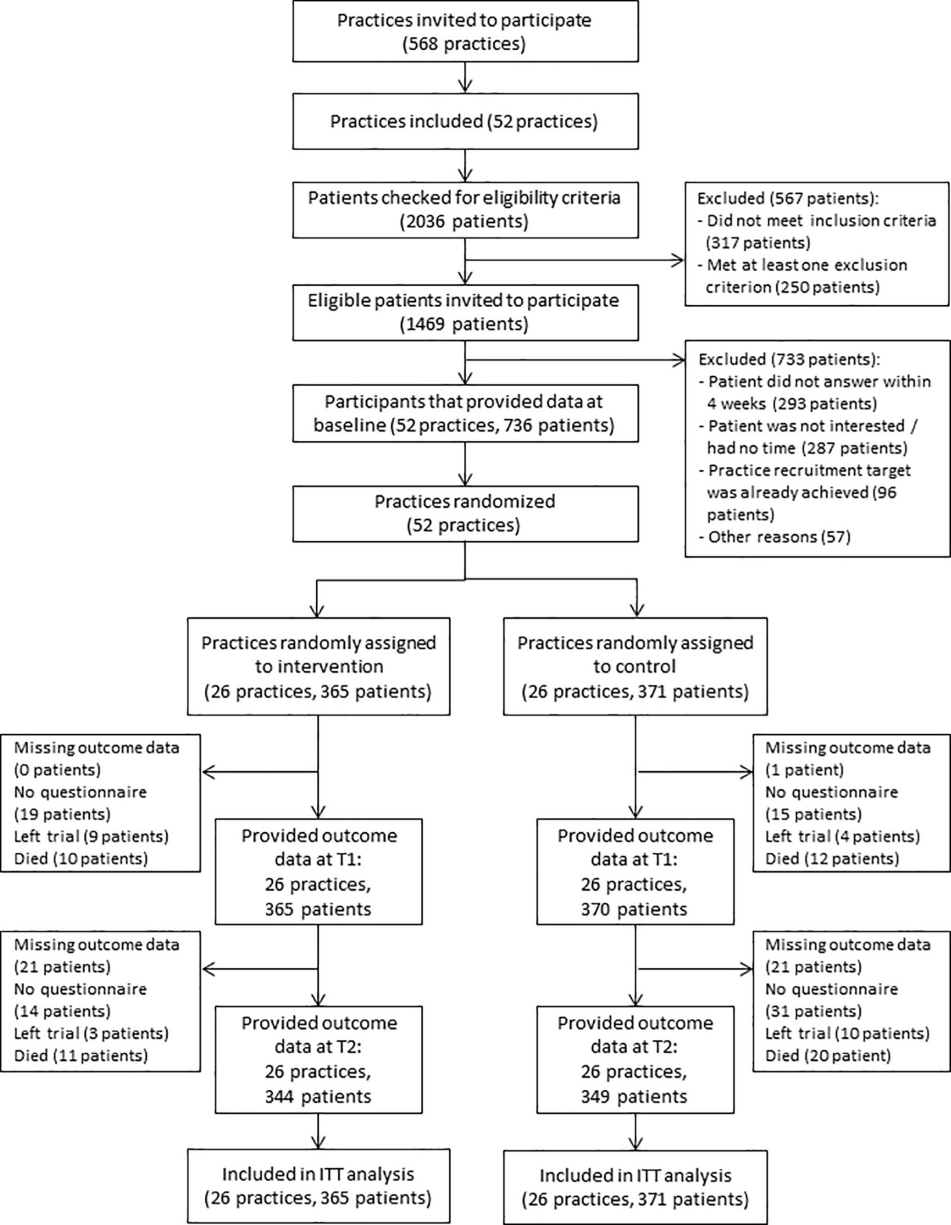
Flow diagram for patients.

The final study sample comprised 736 patients (Fig 1). A comparison of baseline characteristics showed that the groups were similar in terms of practice type (42.3% single-handed practices in each group, see S2 Table). However, a lower proportion of intervention than control practices had third-party certification in quality management procedures (46.2% vs. 65.4%), and fewer of them provided structured courses for patients (42.3% vs. 61.5%).

We enrolled patients between July 2, 2012 and Dec 4, 2012. The mean (SD) number of patients recruited per practice was 14.0 (1.6) in the intervention and 14.3 (1.5) in the control group.

A baseline comparison of enrolled patients and those that were eligible but did not participate (‘non-participants’; n = 733) showed that the groups were similar with regard to age (mean (SD) age was 73.5 (9.4) years in the study population vs. 75.0 (10.9) among non-participants), sex (55.0% of participants were male vs. 52.9% of non-participants), and migration background (6.9% of participants vs. 8.2% of non-participants). More participants (n = 85, 12.3%) than non-participants (n = 52, 8.3%) performed OAC self-management previous to study recruitment.

The ITT and mITT analyses included all patients, compared with 313 intervention and 360 control recipients in the PP analysis.

Sociodemographic and clinical characteristics of patients at baseline were similar in intervention and control groups (Table 1).

**Table 1:** Baseline characteristics of patients.

	Intervention (n = 365)	Control (n = 371)
**Sociodemographic characteristics**		
Age, mean (SD), y^a^	74.4 (9.5)	72.8 (9.3)
Male (sex), no. (%)	205 (56.2)	200 (53.9)
BMI, mean (SD)	28.8 (5.1)	29.1 (4.8)
Migration background, no. (%)	27 (7.4)	24 (6.5)
**Clinical characteristics**		
Long-term indication for oral anticoagulation therapy, no. (%)^b^		
Atrial fibrillation/flutter	302 (82.7)	295 (79.5)
Recurrent venous thromboembolism	32 (8.8)	40 (10.8)
Recurrent pulmonary embolism	31 (8.5)	30 (8.1)
Mechanical heart prosthesis	29 (7.9)	28 (7.5)
Intracardiac thrombus	3 (0.8)	4 (1.1)
Other indication	33 (9.0)	34 (9.2)
CHA_2_DS_2_-VASc-Score, no. (%)^c^		
> 1	292 (97.0)	282 (95.9)
= 1	9 (3.0)	12 (4.1)
Antithrombotic medication, no. (%)^d^		
Phenprocoumon	341 (93.4)	349 (94.1)
Dabigatran	8 (2.2)	4 (1.1)
Rivaroxaban	7 (1.9)	13 (3.5))
Aspirin	4 (1.1)	6 (1.6)
Other	9 (2.5)	3 (0.8)
Last INR within therapeutic target range, no. (%)^e^	240 (69.2)	239 (68.7)
INR self-management, no. (%)^e^	39 (11.3)	46 (13.3)
Patient compliance, no. (%)^f^		
Very good compliance	308 (84.4)	266 (72.1)
Good compliance	51 (14.0)	86 (23.3)
Non-compliant	6 (1.6)	17 (4.6)

^a^Age was calculated from 15/mm/yyyy since the exact birth date was not documented to ensure data privacy.

^b^Patients may have had more than one indication.

^c^Refers to 595 patients with atrial fibrillation/flutter and available data.

^d^Apixaban and edoxaban had not been approved at the time of the baseline assessment.

^e^ Only considers patients receiving phenprocoumon; target INR range as defined by GP.

^f^ As assessed by GP; data available for 369 patients in control group.

**Fig 2:**
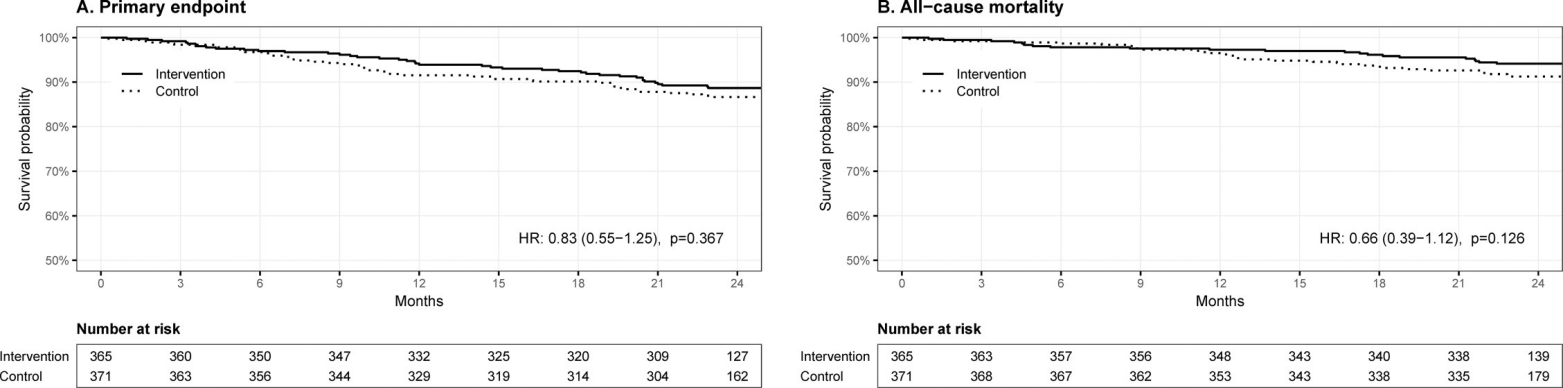
Kaplan-Meier plot of primary outcome and all-cause mortality, ITT analysis.

During the 24-month study period, the primary endpoint occurred in 40 (11.0%) patients in the intervention and 48 (12.9%) patients in the control group (HR 0.83, 95% CI 0.55 to 1.25; *P* = .37) (Table 2). The median (IQR) time-to-event was 355.5 (170–575.5) days in the intervention and 293.5 (174–525.5) days in the control group (Fig 2).

**Table 2:** Intention-to-treat analysis for the primary and key secondary outcomes after 24 months.

	Intervention (n = 365)	Control (n = 371)	ICC	Effect size		95% CI	*P* Value
**Primary outcome**							
Patients suffering a thromboembolic or major bleeding event, no. (%)^a^	40 (11.0)	48 (12.9)	0.00	HR	0.83	(0.55–1.25)	.37
**Key secondary outcomes**							
All-cause mortality, no. (%)	21 (5.8)	32 (8.6)	0.00	HR	0.66	(0.39–1.12)	0.13
Cause-related mortality, no. (%)	4 (1.1)	4 (1.1)	0.00	HR	1.01	(0.28–3.63)	0.98
Number of patients suffering a thromboembolic event, no. (%)^b^	19 (5.2)	26 (7.0)	0.02	OR	0.72	(0.37–1.42)	0.34
Number of patients suffering a major bleeding event, no. (%)^b^	24 (6.6)	25 (6.7)	0.01	OR	0.98	(0.53–1.79)	0.94
Hospitalized patients, no. (%)	184 (50.4)	209 (56.3)	0.00	OR	0.78	(0.59–1.05)	0.099
Number of hospitalizations per patient, median (IQR) ^c^	2 (1–3)	2 (1–4)	0.01	RR	0.88	(0.75–1.03)	0.11
Days of hospitalization per patient, median (IQR)^c^	12 (6–35)	16 (6–35)	0.03	RR	0.89	(0.68–1.17)	0.41
Health-related quality of life (EQ-5D), mean (SD)^d^	-0.03 (0.2)	-0.02 (0.2)	0.00	MD	-0.02	(-0.05, 0.01)	0.27
Number of patients suffering a potentially severe treatment interaction, no. (%)	165 (45.2)	144 (38.8)	0.03	OR	1.29	(0.91–1.84)	0.16
Number of patients suffering an adverse event, no. (%)	85 (23.3)	62 (16.7)	0.17	OR	1.52	(0.75–3.07)	0.25
Time within therapeutic range, mean (SD)^e^	72.5 (18.5)	71.7 (18.1)	0.08	MD	0.73	(-3.18, 4.64)	0.71

^a^If more than one event occurred in a patient, the earliest event was counted.

^b^Counting every event.

^c^Of those patients ever hospitalized.

^d^Changes from baseline to 24 months, n = 590.

^e^Percentage of time within therapeutic range calculated using the Rosendaal algorithm, n = 688.

The ITT analyses of the key secondary outcomes showed no statistically significant difference between groups (see Table 2 and Fig 2). Two patients (1 in each group) experienced a recurrent stroke.

After 24 months, the ITT analyses of further secondary outcomes showed that intervention patients rated their quality of chronic illness care more highly than control patients, with a mean (SD) PACIC score of 6.7 (2.8) vs. 5.9 (2.9)(Table 3). The change from baseline was significantly better (MD 0.87, 95% CI 0.37 to 1.37; *P* <0.001) in the intervention group. Mean (SD) patient knowledge of oral anticoagulation after 24 months was 6.4 (2.9) in the intervention group and 5.5 (2.5) in the control group. Again, changes from baseline were significantly better in the intervention group (MD 0.90, 95% CI 0.44 to 1.36; *P* <0.001). There were no differences in changes from baseline for either patient self-reported adherence to medication, or satisfaction with shared decision-making. The improvement in HCA knowledge of OAC was significantly greater in the intervention group, whereas no difference was found in GP knowledge of OAC (Table 3).

**Table 3:** Intention-to-treat analysis for further secondary outcomes after 24 months.

	Intervention	Control	MD	95% CI	*P* Value
Patient assessment of chronic illness care (PACIC), mean (SD)	0.6 (2.7)	-0.3 (2.7)	0.87	(0.37, 1.37)	<0.001
Patient knowledge about OAC, mean (SD) ^a^	0.6 (2.6)	-0.3 (2.3)	0.90	(0.44, 1.36)	<0.001
Adherence (Morisky), mean (SD)	-0.03 (0.6)	-0.05 (0.7)	0.01	(-0.09, 0.12)	0.82
Satisfaction with shared decision-making (Man-Song Hing test), mean (SD)	0.1 (0.8)	0.2 (0.7)	-0.10	(-0.22, 0.03)	0.13
GP knowledge about OAC, mean (SD)	0.9 (1.6)	0.9 (1.7)	-0.02	(-0.95, 0.91)	0.97
HCA knowledge about OAC, mean (SD)	1.4 (1.1)	0.3 (0.8)	1.08	(0.52, 1.64)	<0.001

All values in this table represent changes from baseline to 24 months.

^a^Results on knowledge have already been published.

No major differences between the two groups regarding the baseline characteristics were observed for the PP. Therefore the same analyses were applied to this population. For the primary endpoint, the PP (Table 4) and modified ITT (S3 Table), as well as the sensitivity (S4 Table) and the subgroup analysis (S5 Table) resulted in similar findings to the ITT analysis. For the key secondary endpoints, the PP analyses showed that statistically significantly fewer intervention (n = 150, 47.9%) than control patients (n = 202, 56.1%) were hospitalized within 24 months (OR 0.72; 95% CI 0.53 to 0.97; *P* = 0.031; Table 4). The number of hospitalizations per patient was also lower among intervention patients (RR 0.85, 95% CI 0.72 to 1.00; *P* = 0.047), whereas the PP analysis yielded similar results to the ITT analysis with regard to the other key secondary outcomes (Table 4).

**Table 4:** Per-protocol analyses for the primary and key secondary outcome after 24 months^a^.

	Intervention (n = 313)	Control (n = 360)	Effect size		95% CI	*P* Value
**Primary outcome**						
Patients suffering a thromboembolic or major bleeding event, no. (%)	30 (9.6)	48 (13.3)	HR	0.70	(0.44–1.09)	0.12
**Key secondary outcomes**						
All-cause mortality, no. (%)	17 (5.4)	30 (8.3)	HR	0.64	(0.36–1.17)	0.15
Cause-related mortality, no. (%)	3 (1.0)	4 (1.1)	HR	0.86	(0.21–3.46)	0.83
Number of patients suffering a thromboembolic event, no. (%)^a^	14 (4.5)	26 (7.2)	OR	0.59	(0.28–1.27)	0.18
Number of patients suffering a major bleeding event, no. (%)^a^	17 (5.4)	25 (6.9)	OR	0.77	(0.40–1.47)	0.43
Hospitalized patients, no. (%)	150 (47.9)	202 (56.1)	OR	0.72	(0.53–0.97)	0.031
Number of hospitalizations per patient, median (IQR)^b^	2 (1–3)	2 (1–4)	RR	0.85	(0.72–1.00)	0.047
Days of hospitalization per patient, median (IQR)^b^	12 (5–32)	15 (6–35)	RR	0.82	(0.61–1.08)	0.16
Health-related quality of life (EQ-5D), mean (SD)^c^	-0.04 (0.2)	-0.02 (0.2)	MD	-0.02	(-0.05, 0.02)	0.27
Number of patients suffering a potentially severe treatment interaction, no. (%)	144 (46.0)	139 (38.6)	OR	1.35	(0.93–1.94)	0.11
Number of patients suffering an adverse event, no. (%)	73 (23.3)	61 (16.9)	OR	1.51	(0.73–3.14)	0.26
Time within therapeutic range, mean (SD)^d^	73.3 (18.3)	71.5 (18.0)	MD	1.65	(-2.36, 5.67)	0.42

^a^As defined for the primary endpoint.

^b^Of those patients ever hospitalized.

^c^Changes from baseline to 24 months, n = 545.

^d^Percentage of time within therapeutic range calculated using the Rosendaal algorithm, n = 637.

The estimated mean cost of the intervention, including the training course for the HCAs and telephone calls for GPs, as well as all HCA and GP contacts (assessments and monitoring), was €215 per patient in the first year and €175 per patient in the second year (S6 Table).

## Discussion

Our trial compared a best-practice model for optimized antithrombotic treatment in patients with a long-term indication for oral anticoagulation to routine care. Even though the main outcomes in the intervention and control groups did not differ significantly, the intervention appears to have positively influenced process parameters such as patients’ perceived quality of care and patient knowledge [20], and HCA knowledge about OAC. For patients obtaining treatment without major protocol deviations (per-protocol analysis), hospital admissions were significantly reduced in the intervention group. Some of our specific intervention elements, such as symptom monitoring and follow up, may have contributed to reduced hospital admissions.

Intervention costs were reasonable and similar to a recent study on HCA-based case management for high-risk patients [21].

To the best of our knowledge, PICANT is the largest trial to date to investigate the effects of a complex intervention involving primary care-based case management, self-management of OAC, and additional patient education. The intervention is feasible in a ‘real world’ setting and does not require additional personnel but rather relies on HCAs as a valuable resource to provide team-based care to chronically ill patients. The professionalization of non-physician health professionals, such as nurses and HCAs, has been identified as a cost-efficient way to improve healthcare [22]. Team-based care approaches and delegation of “transactional tasks” (e.g., documentation of care) to other clinical professionals and staff who have less training means physicians have more time for “personalized” aspects of patient care (e.g., customizing care for individual patients) [23].

Healthcare assistants are a promising resource for delivery of care management to high-risk patients in small primary care practices. Our study aimed to limit the major risks associated with poor anticoagulation control (e.g., long intervals between measurements). Healthcare assistants (supervised by GPs) were able to assume a new role in chronic care management of patients with oral anticoagulation.

In our paper, we restricted our intervention to optimizing antithrombotic management. However, recent studies that examined multidisciplinary integrated care approaches in a patient group with atrial fibrillation, and took multiple co-morbidities into account, showed clear superiority in terms of reducing cardiovascular hospitalization and mortality [24]. Furthermore, an ongoing cluster-RCT is currently seeking to demonstrate the feasibility of integrated atrial fibrillation care in 1000 elderly primary care patients from around 18 to 30 general practices in terms of its potential effectiveness on patient relevant outcomes [25]. Although interesting, this approach goes beyond the scope of our study.

We acknowledge a potential selection bias since a slightly higher percentage of participants than non-participants performed self-management. Participants may therefore have been more highly motivated than the eligible population from which we drew the sample. In addition, both groups already showed fair to good OAC quality at baseline, and in both, the number of patients with INR values within their therapeutic ranges increased further. More of the control practices had third-party certification in quality management than intervention practices. It is therefore probable that oral anticoagulation management in our control group was particularly good, which would have made it more difficult to demonstrate statistically significant differences between the groups. We designed our study in accordance with the recommendations of the extended CONSORT statement [26], so even though we had to deal with certain external constraints, such as limited available funding and the limited duration of the funding period, we consider our results reliable. The intervention intensity was limited so that the additional tasks associated with PICANT would fit into healthcare assistants' daily workflow. However, we over-estimated the anticipated effect of our intervention, and the calculated patient numbers of 317 in each group turned out to be too low. This is a recurrent problem in cluster RCTs, as recently described by Siebenhofer at al. in a methodological systematic review [27]. As our actual ICC was lower than the assumed value, the underestimation of intra-cluster similarities was not a limitation in our study.

The study took place against a background of increasing prescriptions of DOACs. Since receiving approval in 2011, prescriptions of DOACs have risen strongly, with 38 million (m) defined daily doses (DDDs) prescribed in Germany in 2012 (vs. 389m DDDs of VKAs), and 253m DDDs prescribed in 2015 (vs. 346m DDDs of VKAs) [5].

The percentage of VKA patients that switched to DOACs was 7.6% in our trial, lower than the 15% observed in a study by Bleckwenn et al., which was also conducted in German primary care practices [28]. One should bear in mind that the study took place in 2012, when, for example, the enthusiasm of cardiologists for DOACs was not fully shared by GPs. One reason for this was an unfamiliar inability to monitor patients to ensure adherence, while another was cost, since GPs are held responsible for the lion’s share of overall drug costs in Germany. Education and monitoring remain necessary when VKAs are replaced with DOACs. Amara et al. have recently shown that DOAC patients have serious knowledge gaps with respect to their medication [29], with only 21% aware that regular monitoring of renal function is recommended.

In line with the recommendations of evidence-based medicine [30–32], various sources of information have been made available to allow this study to be reproduced. These include the study protocol [11], publications describing the development of the CoMol monitoring list [13], and the screening process [12], as well as information on patient education for self-management [33].

The complex intervention in our study was designed to be provided in addition to routine care of orally anti-coagulated patients with both vitamin K antagonists and newer direct oral anticoagulants. As the anticoagulation of most patients was already of high quality, the assumption that the intervention would improve the long-term outcomes of anticoagulated patients could not be proven. Nevertheless, it should be noted that quality is and remains optimal when primary care professionals have received adequate training. The intervention even led to improved process parameters such as patient knowledge and perceived quality of care, both of which are known to positively influence clinical outcomes and reduce hospital admissions. This was indeed the case in the per-protocol analysis, even though the combined primary outcome of all thromboembolic events requiring hospitalization and all major bleeding complications did not differ significantly between the groups. In addition, supportive team-based care can be provided to chronically ill patients using the publicly available tools we developed (teaching materials, monitoring lists, fact sheets and patient information).

## Supporting information

S1 Table Description of the intervention and control elements. (DOCX)

S2 Table Characteristics of practices, GPs and healthcare assistants. (DOCX)

S3 Table Modified intention-to-treat analysis of the primary and key secondary outcomes after 24 months. (DOCX)

S4 Table Sensitivity analysis for the primary outcome after 24 months. (DOCX)

S5 Table Subgroup analysis by gender for primary and key secondary outcomes after 24 months. (DOCX)

S6 Table Costs. (DOCX)

## Abbreviations

Co-MoL: Coagulation-Monitoring-List
DOACs: direct oral anticoagulants
GP: general practitioner
HCA: healthcare assistant
INR: international normalized ratio
ITT: intention to treat
OAC: oral anticoagulation
OR: odds ratio
PP: per protocol
SD: standard deviation
VKA: vitamin k antagonists
