# Supplementary material for: Primary care management for patients receiving long-term antithrombotic treatment: A cluster-randomized controlled trial
Source: PLoS One. 2019 Jan 9;14(1):e0209366. doi: 10.1371/journal.pone.0209366 (PMC6326474; doi:10.1371/journal.pone.0209366)
Supplement: S1 Table — (DOCX) [file pone.0209366.s001.docx]

**S1 Table. Description of the intervention and control elements.**

|  | **Intervention** | **Control** |
| --- | --- | --- |
| Provision of the evidence-based “Anticoagulation” guideline for general practice^a^ | 🗸 | 🗸 |
| Provision of additional tools and guidelines: patient information leaflet, patient video, fact sheets for phenprocoumon (e.g., Marcumar), dabigatran (Pradaxa), rivaroxaban (Xarelto) | 🗸 | - |
| Interactive, one-day workshop for healthcare assistants | 🗸 | After completion of study |
| Telephone call with every general practitioner before start of intervention | 🗸 | - |
| On average, 9 monitoring sessions per patient in first year and 10 in second year based on “Co-MoL” | 🗸 | - |
| Three quality circles | 🗸 | - |
| Care as usual | - | 🗸 |

^a^ Leitliniengruppe Hessen: Hausärztliche Leitlinie Antikoagulation [Hesse Guideline Group: Anticoagulation in General Practice].
