# Supplementary material for: Primary care management for patients receiving long-term antithrombotic treatment: A cluster-randomized controlled trial
Source: PLoS One. 2019 Jan 9;14(1):e0209366. doi: 10.1371/journal.pone.0209366 (PMC6326474; doi:10.1371/journal.pone.0209366)
Supplement: S2 Table — (DOCX) [file pone.0209366.s002.docx]

**S2 Table. Characteristics of practices, GPs and healthcare assistants.**

| **Characteristics of practices** | **Intervention**  (n = 26) | **Control**  (n = 26) |
| --- | --- | --- |
| Single-handed practice, no. (%) | 11 (42.3) | 11 (42.3) |
| Third-party certification in quality management  for medical practices (e.g., QEP) ^a^ | 12 (46.2) | 17 (65.4) |
| Rural location, no. (%)^b^ | 12 (46.2) | 10 (38.5) |
| Panel size, registered patients per quarter, no. (%)^c^ |  |  |
| 500-999 | 7 (26.9) | 1 (3.8) |
| 1000-1499 | 9 (34.6) | 11 (42.3) |
| 1500-1999 | 7 (26.9) | 5 (19.2) |
| ≥ 2000 | 3 (11.5) | 9 (34.6) |
| Structured training courses for patients, no. (%) | 11 (42.3) | 16 (61.5) |
| **Characteristics of GPs** |  |  |
| Sex (male), no. (%) | 18 (69.2) | 16 (61.5) |
| Age, mean (SD) | 52.4 (7.7) | 49.3 (7.4) |
| Years of job experience since medical school, mean (SD) | 23.1 (8.1) | 20.4 (7.9) |
| Participated in a study in the last 5 years, no. (%)^d^ | 8 (30.8) | 11 (42.3) |
| **Characteristics of healthcare assistants** |  |  |
| Age, mean (SD) | 40.4 (11.8) | 37.9 (12.4) |
| Years of job experience (including education), mean (SD) | 19.3 (10.1) | 18.6 (11.7) |

^a^The quality management system QEP (Qualität und Entwicklung in Praxen [Quality and Development in practices]) was developed by the National Association of Statutory Health Insurance Physicians and regional Associations of Statutory Health Insurance Physicians.

^b^Rural refers to a region or town with <20,000 inhabitants.

^c^In Germany, panel size is calculated as the number of patient registrations in a practice over a three-month period.

^d^Including studies conducted by our own Institute and others (e.g., pharmaceutical companies).
