# Supplementary material for: Primary care management for patients receiving long-term antithrombotic treatment: A cluster-randomized controlled trial
Source: PLoS One. 2019 Jan 9;14(1):e0209366. doi: 10.1371/journal.pone.0209366 (PMC6326474; doi:10.1371/journal.pone.0209366)
Supplement: S3 Table — (DOCX) [file pone.0209366.s003.docx]

**S3 Table. Modified intention-to-treat analysis of the primary and key secondary outcomes after 24 months**^a^.

|  | **Intervention  (n=365)** | **Control**  **(n=371)** | **HR** | **95% CI** | ***P* Value** |
| --- | --- | --- | --- | --- | --- |
| **Primary outcome** |  |  |  |  |  |
| Patients suffering a thromboembolic or major bleeding event, no. (%)^b^ | 38 (10.4) | 46 (12.4) | 0.81 | (0.54-1.20) | 0.29 |
| **Key secondary outcomes** |  |  |  |  |  |
| All-cause mortality, no. (%) | 19 (5.2) | 29 (7.8) | 0.64 | (0.38-1.10) | 0.11 |
| Cause-related mortality, no. (%) | 4 (1.1) | 3 (0.8) | 1.34 | (0.33-5.42) | 0.69 |

^a^Patients switching to a DOAC were censored at the time of switching.

^b^If more than one event occurred in a patient, only the earliest event was considered.
