# Supplementary material for: Primary care management for patients receiving long-term antithrombotic treatment: A cluster-randomized controlled trial
Source: PLoS One. 2019 Jan 9;14(1):e0209366. doi: 10.1371/journal.pone.0209366 (PMC6326474; doi:10.1371/journal.pone.0209366)
Supplement: S4 Table — (DOCX) [file pone.0209366.s004.docx]

**S4 Table. Sensitivity analysis for the primary outcome after 24 months**^a^.

|  | **Intervention  (n = 365)** | **Control (n = 371)** | **HR** | **95% Confidence interval** | ***P* Value** |
| --- | --- | --- | --- | --- | --- |
| Patients suffering a thromboembolic or major bleeding event, no. (%)^b^ | 39 (10.7) | 46 (12.4) | 0.84 | (0.56-1.26) | 0.40 |

^a^ The intervention date for staff at each intervention practice was used as an alternative start date for this sensitivity analysis. The start date was pushed back 27 days in the control group practices, as this was the median time before the intervention began in the intervention group.

^b^If more than one event occurred in a patient, only the earliest event was considered.
