# Supplementary material for: Primary care management for patients receiving long-term antithrombotic treatment: A cluster-randomized controlled trial
Source: PLoS One. 2019 Jan 9;14(1):e0209366. doi: 10.1371/journal.pone.0209366 (PMC6326474; doi:10.1371/journal.pone.0209366)
Supplement: S5 Table — (DOCX) [file pone.0209366.s005.docx]

**S5 Table. Subgroup analysis by gender for primary and key secondary outcomes after 24 months**

|  | **Gender** | **Intervention  (n = 365)** | **Control**  **(n = 371)** | **Effect size** | | **95% CI** | ***P* Value** |
| --- | --- | --- | --- | --- | --- | --- | --- |
| **Primary outcome** |  |  |  |  |  |  |  |
| Patients suffering a thromboembolic or major bleeding event, no. (%)^a^ | Male | 24 (11.7) | 26 (13.0) | HR | 0.91 | (0.56-1.46) | 0.68 |
|  | Female | 16 (10.0) | 22 (12.9) |  | 0.74 | (0.40-1.36) | 0.33 |
| **Key secondary outcomes** |  |  |  |  |  |  |  |
| All-cause mortality, no. (%) | Male | 15 (7.3) | 18 (9.0) | HR | 0.82 | (0.45-1.49) | 0.52 |
|  | Female | 6 (3.8) | 14 (8.2) |  | 0.44 | (0.17-1.18) | 0.10 |
| Cause-related mortality, no. (%) | Male | 3 (1.5) | 2 (1.0) | HR | 1.48 | (0.26-8.31) | 0.66 |
|  | Female | 1 (0.6) | 2 (1.2) |  | 0.52 | (0.05-5.41) | 0.58 |
| Number of patients suffering a thromboembolic event, no. (%)^b^ | Male | 13 (6.3) | 13 (6.5) | OR | 0.98 | (0.42-2.26) | 0.96 |
|  | Female | 6 (3.8) | 13 (7.6) |  | 0.47 | (0.17-1.30) | 0.15 |
| Number of patients suffering a major bleeding event, no. (%)^b^ | Male | 14 (6.8) | 14 (7.0) | OR | 0.97 | (0.45-2.10) | 0.95 |
|  | Female | 10 (6.3) | 11 (6.4) |  | 0.97 | (0.40-2.36) | 0.95 |
| Hospitalized patients, no. (%) | Male | 100 (48.8) | 107 (53.5) | OR | 0.83 | (0.55-1.27) | 0.40 |
|  | Female | 84 (52.5) | 102 (59.6) |  | 0.74 | (0.48-1.14) | 0.17 |
| Number of hospitalizations per patient, median (IQR)^c^ | Male | 2 (1-3) | 2 (1-4) | RR | 0.90 | (0.74-1.11) | 0.33 |
|  | Female | 2 (1-3) | 2 (1-4) |  | dnc |  |  |
| Days of hospitalization per patient, median (IQR)^c^ | Male | 11.5 (4.5-35) | 13 (4-31) | RR | 1.22 | (0.81-1.84) | 0.34 |
|  | Female | 12 (7-34) | 23 (8-43) |  | 0.74 | (0.53-1.02) | 0.066 |
| Health-related quality of life (EQ-5D), mean (SD)^d^ | Male | -0.04 (0.2) | 0.00 (0.2) | MD | -0.04 | (-0.08, -0.00) | 0.040 |
|  | Female | -0.03 (0.3) | -0.04 (0.2) | MD | 0.01 | (-0.04, 0.07) | 0.69 |
| Number of patients suffering a potentially severe treatment interaction, no. (%) | Male | 82 (40.0) | 82 (41.0) | OR | 0.95 | (0.61-1.48) | 0.83 |
|  | Female | 83 (51.9) | 62 (36.3) |  | 1.85 | (1.14-3.01) | 0.013 |
| Number of patients suffering an adverse event, no. (%) | Male | 48 (23.4) | 32 (16.0) | OR | 1.52 | (0.72-3.20) | 0.27 |
|  | Female | 37 (23.1) | 30 (17.5) |  | 1.58 | (0.66-3.82) | 0.31 |
| Time within therapeutic range, mean (SD) | Male | 73.9 (19.1) | 72.1 (18.4) | MD | 1.85 | (-2.88, 6.57) | 0.44 |
|  | Female | 70.6 (17.6) | 71.4 (17.8) |  | -0.84 | (-5.78, 4.11) | 0.74 |

dnc = did not converge.

^a^If more than one event occurred in a patient, only the earliest event was considered.

^b^Counting every event.

^c^Of those patients ever hospitalized.

^d^Changes from baseline to 24 months.
