# Supplementary material for: Primary care management for patients receiving long-term antithrombotic treatment: A cluster-randomized controlled trial
Source: PLoS One. 2019 Jan 9;14(1):e0209366. doi: 10.1371/journal.pone.0209366 (PMC6326474; doi:10.1371/journal.pone.0209366)
Supplement: S6 Table — (DOCX) [file pone.0209366.s006.docx]

**S6 Table. Costs**

| **Category** | **Mean costs (SD)**^a^ | |
| --- | --- | --- |
|  | **1st year** | **2nd year** |
| **Intervention training**^b^ |  |  |
| GP | 4 | - |
| HCA | 13 | - |
| **Patient assessment per session (one session per year)** |  |  |
| GP | 28 | 21 |
| HCA | 10 | 5 |
| **Patient monitoring per session**^c^ |  |  |
| GP | 9 | 9 |
| HCA | 8 | 6 |
| **Sum of total intervention costs per patient** | **215**^d^ | **175**^d^ |

^a^The intervention costs were estimated using equivalent hourly wages for healthcare assistants (€15.25) and GPs (€54.62). The intervention costs decreased in the second year because the cost of training case managers was only relevant at the beginning of the intervention. The results of cost-effectiveness analyses will be published in more detail separately.

^b^ One-off qualification costs in first year only. The mean training costs totaled €182 for HCAs and €56 for GPs, divided by an average caseload of 14 patients per practice.
^c^ On average, 9 monitoring sessions per patient in first year and 10 monitoring sessions in second year.

^d^ Minor differences are due to rounding.
